# Observations on the Oxalic Diathesis, and the Influence of the Rhubarb Plant in Its Production

**Published:** 1847-01

**Authors:** John S. Bartrum

**Affiliations:** Surgeon, Bath


*Observations on the Oxalic Diathesis, and the Influence of the Rhubarb Plant in its Production.* By JOHN S. BARTRUM, Esq., Surgeon, Bath.—Having for some years been in the habit of paying attention to the general and chemical conditions of the urine, it was with much pleasure that I perused the paper of Mr. Wilson, inserted in No. 35 of the *Provincial Journal*, (September 2, 1846,) especially from having, on several occasions within these two or three last years, noted in my own person, the effects on the urine of the rhubarb stalk and other articles of diet. The portion of urine passed after rising in the morning was always examined by the miscroscope without heat, as the shortest and not fallacious mode.

Being apparently in the most perfect health, excepting rather overworked, and as far as unlike a patient suffering from oxaluria as possible, at first I could scarcely believe myself to have passed oxalate crystals, till confirmed in my supposition by Dr. William Budd, who immediately remarked that I must have been eating rhubarb, which, however, had not been recently the case. This induced further and oft repeated examinations, the general results of which I can only now give, having not long since destroyed the daily records, considering them as not worth keeping.

While passing the smaller oxalates, and then partaking freely of rhubarb, the first effect was generally to increase the size and quantity of oxalates thrown down, with the occasional addition of some of the reniform bodies; the diet being continued, the crystals of all shapes increased in size, especially the latter, till on two occasions they almost solely were passed. However, after a day or two, the oxalates diminished and then disappeared, although rhubarb was still partaken of ; this may probably be explained by having regarded the appearance of the oxalates, as sure warning that I must give myself more relaxation. The results have been similar on two or three occasions, when from continuous exertions I have expected their presence and found them ; but I have never been able to induce their appearance by the freest use of rhubarb, except in the very fine cuboid forms, for a day or so, unless they were previously present.

If Mr. Wilson continue his inquiries, I think he will find, that any article of diet, adding to the irritability of the kidneys and bladder, will induce an additional secretion of the oxalates; for I always found, that partaking freely of water-cresses added materially to the quantity of oxalates, provided I were already passing them, from the irritation of the bladder, caused by the abundant secretion of free lithic acid. Whether the elimination of oxalates, as well as of lithic acid, was due to the water-cresses, or the common salt accompanying them, I cannot say. That must be left for future investigation.

It will be found almost universally, that cases characterised chiefly by deposits of oxalates are combined with an asthenic condition of the assimilating organs ; some of them corresponding closely with those caused by the excretion of an abnormal amount of urea, the urine then being dense and loaded with lithates, while others, from the excessive excretion give rise to a suspicion of diabetes mellitus, so much so, that of the many specimens sent me for examination, where a large quantity of urine is voided, the greater proportion are of low specific gravity, with some few small oxalates, and often swarms of vibriones. These latter cases, however, are essentially cases of want of nervous power without any specific ailment, sometimes passing phosphates, sometimes oxalates, according to the varying condition of the system, which always displays a very reduced vitality.

In this, as in all other diseases of the urinary organs, where chemistry is called into our aid, there is great danger of their being treated as though the body were a mere laboratory, wherein we could modify these secretions at our will, overlooking the essential cause of the ailment; but, however useful and necessary such examinations may be, it behoves the practitioner carefully to eschew being implicitly guided by them; as some cases of this disease are treated successfully by alkalies, others by acids; some by restricted, others by a generous diet; for though medicinal remedies are most useful, and in the majority of cases requisite, they will be of little avail, if not well supported by carefully-applied general and dietetic measures.

I think that the size of the crystals of oxalates passed will often afford a good indication of the extent of the oxalic diathesis. If the crystals, especially the reniform, be large, distinct, much inclined to become clustered, crystalize on the hairs, &c., much oxalate of lime is passing. As the case improves, the crystals lessen in size and numbers till at length they become undistinguishable, except to the educated eye. Though it is unusual, the reniform bodies may continue to the last, when, in some positions, they may become in appearance almost like a blood-corpuscle; in the majority of cases these crystals are not to be found.

It has been suggested (by whom originally I cannot learn,) that these reniform bodies are not oxalate of lime, but lithic acid, modified in shape by the presence of oxalic acid. This can scarcely be, for I have, with several different specimens, macerated the whole deposit in liquor potassæ, to get rid of any free lithic acid; then, in diluted acetic or muriatic acid, whereby the phosphates and lithates are separated; yet both the cuboid and reniform crystals have remained quite unaltered. I have not succeeded in throwing down crystals of oxalate of lime, when a deposit of free lithic acid and oxalates was dissolved in sulphuric acid, and the former separated by the addition of water, perhaps from sufficient care not having been taken in the matter.

Should it be desired to separate any deposit of these salts for examination, it is most easily effected by decanting the upper layers of fluid, adding distilled water to the remainder, with or without potass, or acetic acid. The oxalates soon fall to the bottom, and may be readily collected on a watch-glass, without heat or any other process that could modify its composition after leaving the body. By careful manipulation you may obtain and weigh all the crystallized salt in a given specimen. To those not conversant with the salt naturally deposited, yet desirous to examine it, one of the best modes of learning all its usual shapes and sizes is to add a dilute solution of oxalic acid to fresh healthy urine, when after some hours the characteristic crystals will be found in abundance.

I do not know whether this city more abounds with cases of this disease than most others, but among its labouring population, of the class next above the poor, such as policemen, schoolmasters, carpenters, &c., (some hundreds of whom come wholly under my observation,) dyspepsia, of an atonic character, and marked by the pallid, depressed, emaciated countenance, with more or less hypochondriasis, pain of the side, (often of great intensity,) or of the back, and the passage of oxalate crystals is most rife, though in most cases readily amenable to judicious treatment. From my own observation this form of dyspepsia does not seem so common among the women of this class as among the men, even in those, wherein from their appearance and symptoms I had fully expected to find it. Of the presence of the oxalates in the more acute or in cutaneous diseases, I can say nothing, not having examined them for that purpose.—*Provincial Med. and Sur. Journal.*

